# Cluster Analysis of Obesity and Asthma Phenotypes

**DOI:** 10.1371/journal.pone.0036631

**Published:** 2012-05-11

**Authors:** E. Rand Sutherland, Elena Goleva, Tonya S. King, Erik Lehman, Allen D. Stevens, Leisa P. Jackson, Amanda R. Stream, John V. Fahy, Donald Y. M. Leung for the Asthma Clinical Research Network

**Affiliations:** 1 Department of Medicine, National Jewish Health, Denver, Colorado, United States of America; 2 Department of Pediatrics, National Jewish Health, Denver, Colorado, United States of America; 3 Department of Public Health Sciences, Pennsylvania State University College of Medicine, Hershey, Pennsylvania, United States of America; 4 Department of Medicine, University of Colorado, Denver, Colorado, United States of America; 5 Department of Medicine, University of California San Francisco, San Francisco, California, United States of America; 6 Department of Medicine, University of Colorado, Denver, Colorado, United States of America; Vanderbilt University Medical Center, United States of America

## Abstract

**Background:**

Asthma is a heterogeneous disease with variability among patients in characteristics such as lung function, symptoms and control, body weight, markers of inflammation, and responsiveness to glucocorticoids (GC). Cluster analysis of well-characterized cohorts can advance understanding of disease subgroups in asthma and point to unsuspected disease mechanisms. We utilized an hypothesis-free cluster analytical approach to define the contribution of obesity and related variables to asthma phenotype.

**Methodology and Principal Findings:**

In a cohort of clinical trial participants (n = 250), minimum-variance hierarchical clustering was used to identify clinical and inflammatory biomarkers important in determining disease cluster membership in mild and moderate persistent asthmatics. In a subset of participants, GC sensitivity was assessed via expression of GC receptor alpha (GCRα) and induction of MAP kinase phosphatase-1 (MKP-1) expression by dexamethasone. Four asthma clusters were identified, with body mass index (BMI, kg/m^2^) and severity of asthma symptoms (AEQ score) the most significant determinants of cluster membership (F = 57.1, p<0.0001 and F = 44.8, p<0.0001, respectively). Two clusters were composed of predominantly obese individuals; these two obese asthma clusters differed from one another with regard to age of asthma onset, measures of asthma symptoms (AEQ) and control (ACQ), exhaled nitric oxide concentration (F_E_NO) and airway hyperresponsiveness (methacholine PC_20_) but were similar with regard to measures of lung function (FEV_1_ (%) and FEV_1_/FVC), airway eosinophilia, IgE, leptin, adiponectin and C-reactive protein (hsCRP). Members of obese clusters demonstrated evidence of reduced expression of GCRα, a finding which was correlated with a reduced induction of MKP-1 expression by dexamethasone

**Conclusions and Significance:**

Obesity is an important determinant of asthma phenotype in adults. There is heterogeneity in expression of clinical and inflammatory biomarkers of asthma across obese individuals. Reduced expression of the dominant functional isoform of the GCR may mediate GC insensitivity in obese asthmatics.

## Introduction

Cluster analyses of cross-sectional data from clinical populations have identified phenotypic subsets of patients with asthma, and the assessment of BMI in recent asthma cluster analyses has allowed assessment of the relationship of BMI to clinical features of asthma. Haldar and colleagues reported that obesity was associated with increased symptom expression, reduced eosinophilic airway inflammation, adult age of onset, and female sex, while also being associated with reduced clinical responsiveness to inhaled corticosteroids (ICS) [1]. A separate cluster analysis of patients participating in the NIH Severe Asthma Research Program indicated that elevated body mass index (BMI) was associated with specific clinical features in severe asthma, with the identification of a cluster of patients in whom elevated BMI was associated with female sex, adult onset asthma, a greater likelihood of complicated asthma treatment regimens, and more frequent health care utilization and need for systemic glucocorticoids (GC) [2]. These two studies have supported the conclusion that asthma phenotype is relatively homogenous in obese patients, with high symptom expression, low atopy and airway eosinophilia, and relative insensitivity to GC, a phenomenon that has been reported by others in both clinical [3]–[5] and *in vitro* [6] settings.

These reports notwithstanding, other analyses using standard comparative analytical approaches between asthmatics categorized by BMI have suggested that there is phenotypic heterogeneity among obese asthmatics, with some studies suggesting that asthma is more severe in obese asthmatics [7], [8], possibly due to increased airway or systemic inflammation [9], and others suggesting that obesity has a more modest effect, independent of inflammation or altered lung mechanics [10], [11]. Given these disparate observations, and given that over 50% of adult asthmatics are overweight or obese [12], more information is needed to enhance our understanding of asthma in obese patients. In this context, we conducted an analysis of data from two NIH-sponsored clinical trials to evaluate the relationship of obesity to markers of asthma phenotype in adults. *A priori*, we incorporated measurements of adipokines and systemic inflammation and clinical measures of response to inhaled corticosteroids into a cluster-based analytical approach. We then evaluated differences in *in vitro* markers of glucocorticoid sensitivity between phenotype clusters identified in the hypothesis-independent analysis.

## Methods

### Objective

To define the contribution of obesity and related variables to asthma phenotype.

### Participants

Data from adults with persistent asthma participating in the common run-in period of the TALC [13] (NCT00565266) and BASALT trials (NCT00495157) of the NHLBI Asthma Clinical Research Network were utilized to assemble a cohort of rigorously characterized asthmatic subjects. Participants at all centers were assessed for biomarkers of obesity and systemic inflammation. Inclusion/exclusion criteria and design of the common run-in period have been reported previously [13]. Of the 826 participants enrolled in the common run-in period, all participants with complete clinical, physiologic and inflammatory data (n = 250) were eligible for inclusion in these analyses.

### Description of Procedures

During the common run-in period, all participants received hydrofluoroalkane beclomethasone dipropionate (HFA-BDP) at a dose of 80 mcg (2 puffs of 40 mcg) twice daily for a 4-week period and were provided an albuterol metered-dose inhaled for rescue use. Clinical and inflammatory parameters were assessed as reported previously [13]. Peripheral blood mononuclear cells (PBMC) were isolated by Ficoll-Hypaque gradient centrifugation [14] and cultured in medium ± dexamethasone (DEX, 10^−6^ M) for 3 hours. RNA was extracted and expression of GC receptor-alpha (GCRα) and MAP kinase phosphatase-1 (MKP-1) were analyzed by real-time PCR, normalized to corresponding levels of 18 sRNA, and reported as fold-change in expression induced by dexamethasone [15], [16]. 25(OH)D concentrations were assayed by liquid chromatography-tandem mass spectrometry at Mayo Clinical Laboratories (Rochester, Minnesota). Assays for serum concentrations of leptin, adiponectin, interleukin 6 (IL-6), tumor necrosis factor-alpha (TNFα) and C-reactive protein were performed by ELISA or highly-sensitive immunoturbidometric assay (hsCRP).

### Ethics

All participants provided written informed consent. The protocol was reviewed and approved at each institutional IRB listed in the Appendix S1.

### Statistical Methods

Ward’s minimum-variance hierarchical clustering method [17] with standardization of incorporated variables was performed in SAS (v. 9.2, SAS Institute Inc., Cary, N.C.). Analyzed variables included sex, race (white versus nonwhite), age at asthma onset, asthma duration, body mass index (BMI), % predicted forced expiratory volume in one second (FEV_1%_), forced vital capacity (FVC), airway hyperresponsiveness (PC_20_ FEV_1_ to methacholine (mg/mL)), Juniper Asthma Control Questionnaire score (ACQ) [18], Asthma Evaluation Questionnaire score (AEQ, a composite of asthma symptoms over the prior two weeks [13]), exhaled nitric oxide (F_E_NO, ppb), percent eosinophils in induced sputum, serum IgE (IU/mL), hsCRP, serum IL-6, serum TNFα, serum adiponectin, serum leptin, prior controller use, and change in AEQ and ACQ scores (after 4 and 2 weeks, respectively, of HFA-BDP). Discriminant analysis was then performed to identify significant determinants of cluster membership, and a reclassification procedure determined the accuracy of the discriminant function model for predicting cluster membership. Generalized squared distances were utilized to determine the proximity of clusters.

Differences between clusters were evaluated using analysis of variance or Student’s t-test for normally-distributed continuous variables. Chi-square analysis was used for categorical measures. Non-normally distributed data were log-transformed for analysis. Unadjusted analyses correlating continuous variables were performed using simple linear regression, with least-squares regression was used to perform adjusted analyses. Numeric data are presented as mean (standard deviation), except in the case of geometric mean (coefficient of variation) for log-transformed data.

## Results

### Participant Characteristics

Data from 250 participants were analyzed (Table 1). The population was 32% male, 59% white and had a mean (SD) age of 37.6 (12.5) years. The study population had a mean BMI of 29.9 (8.3) kg/m^2^, with a mean FEV_1_ of 82.2 (13.8) % predicted and airway hyperresponsiveness as reflected by a methacholine PC_20_ FEV_1_ of 1.2 (1.2) mg/mL. Serum IgE was 105.4 (1.6) IU/mL, F_E_NO was 19.9 (0.6) ppb, and sputum eosinophils were 0.8 (1.0)% (geometric mean and coefficient of variation).

**Table 1:** Characteristics of study population.

Measured at study initiation	
n, subjects	250
Sex (% male)	32
Race (% white)	59
Age (years)	37.6 (12.5)
Age of asthma onset (years)	15.4 (14.7)
Asthma duration (years)	22.2 (12.2)
BMI (kg/m^2^)	29.9 (8.3)
FEV_1_ (L)	2.8 (0.8)
FVC (L)	3.9 (1.1)
FEV1/FVC (%)	71.8 (8.7)
FEV_1_ (% predicted)	82.2 (13.8)
PC_20_ (mg/mL)†	1.2 (1.2)
Asthma Evaluation Questionnaire Score	0.7 (0.8)
Measured after 2 weeks HFA-BDP	
Asthma Control Questionnaire Score	1.0 (0.8)
IgE (IU/mL) †	105.4 (1.6)
hsCRP (mg/L )†	1.8 (1.4)
Interleukin-6 (pg/mL)†	1.4 (0.9)
TNFα(pg/mL)†	1.7 (0.8)
Adiponectin (mcg/mL)†	7.0 (0.7)
Leptin (ng/mL)†	10.8 (1.3)
Measured after 4 weeks HFA-BDP	
F_E_NO (ppb) †	19.9 (0.6)
Sputum eosinophils (%) †	0.8 (1.0)
Asthma Evaluation Questionnaire Score	0.6 (0.7)
Asthma Control Questionnaire Score	0.9 (0.8)

Numeric data presented as mean (standard deviation), except ^†^geometric mean (coefficient of variation), log-transformed for analysis.

### Determinants of Cluster Membership

Discriminant analysis revealed that 16 variables (Table 2) were significant determinants of cluster membership, with reclassification indicating that the discriminant function model achieved 89% accuracy for predicting cluster membership. BMI was the most significant determinant of cluster membership (F = 57.1, p<0.0001), followed by asthma symptoms (F = 44.8, p<0.0001). Less significant were degree of asthma control (ACQ, F = 12.5, p<0.0001), race (F = 9.4, p<0.0001), degree of improvement in asthma symptoms after 4 weeks of treatment with HFA-BDP (F = 9.1, p<0.0001), age of onset/disease duration, lung function, airway hyperresponsiveness (PC_20_), leptin, adiponectin, biomarkers of systemic (hsCRP, TNFα), airway inflammation (F_E_NO) and atopy (IgE). Generalized squared distances between the clusters ranged from 7.8 to 16.0, with pair-wise differences as follows: cluster 1 vs. 2, 9.2; cluster 1 vs. 3, 15.3; cluster 1 vs. 4, 7.8; cluster 2 vs. 3, 16.0; cluster 2 vs. 4, 13.1; and cluster 3 vs. 4, 13.3.

**Table 2:** Results of discriminant analysis demonstrating relative contribution of variables in determining cluster membership.

Variable	Partial R-Square	F	p
BMI	0.4105	57.1	<.0001
AEQ (symptoms)	0.3542	44.8	<.0001
ACQ (control)	0.1339	12.5	<.0001
Race	0.1039	9.4	<.0001
Change in AEQ after 4 weeks of HFA-BDP	0.1021	9.1	<.0001
Age of asthma onset	0.0991	8.8	<.0001
F_E_NO	0.0845	7.4	<.0001
Asthma controller type	0.0724	6.2	0.0005
FEV_1%_ predicted	0.0696	5.9	0.0007
Leptin	0.0651	5.5	0.0012
Asthma duration	0.0630	5.2	0.0017
Adiponectin	0.0601	5.0	0.0022
TNFα	0.0587	4.9	0.0027
PC_20_	0.0474	3.9	0.0100
IgE	0.0385	3.1	0.0282
FVC	0.0358	2.9	0.0372

### Asthma Clusters

Analysis revealed four unique clusters of asthma patients, with characteristics as reported in Table 3. These four clusters differed from each other significantly with regard to BMI, with mean BMI in clusters 1 and 2 falling within the overweight range (BMI = 25.8 (5.0) and 26.9 (4.4)). In contrast, BMI in clusters 3 and 4 was indicative of class I and class II obesity [19] (34.7 (8.0) and 38.5 (9.2), respectively), p<0.01 for comparison between the four clusters. As shown in Table 3, FEV_1_ was highest and IgE, hsCRP and leptin were all lower in the non-obese clusters when compared with the two obese clusters. All clusters were marked by low sputum eosinophils and did not differ significantly from each other (Table 3). Concentrations of hsCRP were highest in the two obese clusters, with hsCRP concentration of 4.2 (1.2) and 4.5 (1.1) in clusters 3 and 4 and 1.3 (1.3) and 0.8 (1.1) mg/L in clusters 1 and 2.

**Table 3:** Characteristics of asthma disease clusters.

	Nonobese female asthmatics	Nonobese male asthmatics	Obese uncontrolled asthma	Obese well-controlled asthma	p
Cluster number	1	2	3	4	-
n	114	52	30	54	-
Sex (% male)	18	83	17	24	<0.01
Race (% white)	77	67	37	26	<0.01
Age at onset (years)	19.1 (16.1)	9.8 (11.8)	10.0 (10.8)*	16.1 (13.9)*	<0.01
Asthma duration (years)	18.3 (11.3)	26.2 (11.5)	25.9 (12.0)	24.6 (12.9)	<0.01
BMI (kg/m^2^)	25.8 (5.0)	26.9 (4.4)	34.7 (8.0)	38.5 (9.2)	<0.01
FVC (L)	3.8 (0.7)	4.9 (1.3)	3.2 (0.9)	3.3 (0.9)	<0.01
FEV_1_ (% predicted)	87.7 (12.1)	82.3 (16.4)	73.5 (9.0)	75.5 (11.1)	<0.01
FEV1/FVC (%)	74.1 (8.7)	68.5 (8.7)	71.5 (8.0)	69.7 (8.0)	<0.01
PC_20_, mg/mL†	1.2 (1.2)	1.6 (1.3)	0.7 (1.2)*	1.5 (0.9)*	0.02
ACQ Score	0.8 (0.7)	0.8 (0.6)	1.8 (1.0)*	0.9 (0.9)*	<0.01
AEQ Score	0.5 (0.6)	0.4 (0.5)	1.3 (0.9)*	0.7 (0.8)*	<0.01
F_E_NO (ppb) †	20.8 (0.6)	21.6 (0.6)	24.8 (0.7)*	14.9 (0.7)*	<0.01
Eosinophils (%) †	0.8 (0.9)	0.9 (1.0)	0.8 (1.1)	0.7 (0.9)	0.44
IgE (IU/mL) †	78.1 (1.7)	99.8 (1.3)	201.9 (1.5)	146.1 (1.4)	<0.01
hsCRP (mg/L )†	1.3 (1.3)	0.8 (1.1)	4.2 (1.2)	4.5 (1.1)	<0.01
Interleukin-6 (pg/mL)†	1.2 (1.0)	0.9 (0.6)	1.9 (0.7)	2.1 (0.7)	<0.01
TNFα(pg/mL)†	2.0 (1.0)	1.4 (0.4)	1.4 (0.6)	1.5 (0.7)	0.03
Adiponectin (mcg/mL)†	10.2 (0.6)	4.8 (0.6)	6.3 (0.7)	4.9 (0.7)	<0.01
Leptin (ng/mL)†	9.3 (1.0)	3.4 (1.3)	23.1 (0.9)	29.3 (0.8)	<0.01
Use of medium/high-dose ICS (%)	26	21	37	43	0.06

Table p values from Pearson chi-square test (Exact or CMH test) or analysis of variance comparing all 4 clusters.

* indicates p<0.05 for comparison of clusters 3 and 4.

Numeric data presented as Mean (Standard Deviation), except.

† Geometric Mean (Coefficient of Variation), log-transformed for analysis.

ACQ: asthma control questionnaire score after 4 weeks of HFA-BDP, AEQ: asthma evaluation questionnaire score after 4 weeks of HFA-BDP.

### Phenotypic Heterogeneity in Obese Asthmatics

As reported in Table 3, obese clusters 3 and 4 were similar with regard to lung function, sex distribution, racial composition, age and concentrations of the adipokines leptin and adiponectin and hsCRP, a marker of systemic inflammation. There was a trend toward a significant BMI difference between the two obese clusters that did not achieve statistical significance, with cluster 3 demonstrating an average BMI of 34.7 (8.0) kg/m^2^, versus 38.5 (9.2) kg/m^2^ in cluster 4 (p = 0.06). Age of asthma onset differed between the two clusters with members of cluster 3 having asthma onset during childhood at 10.0 (10.8) years of age, and members of cluster 4 having disease onset during adolescence, at 16.1 (13.9) years of age. The two obese clusters differed with regard to degree of symptom expression and asthma control despite 4 weeks’ treatment with HFA-BDP: cluster 3 demonstrated persistently high symptom expression, with an AEQ score of 1.3 (0.9) versus 0.7 (0.8) (p<0.01), and also demonstrated persistently worse asthma control, with an ACQ score of 1.8 (1.0) versus 0.9 (0.9) (p<0.01). Cluster 3 also demonstrated the highest concentration of F_E_NO at 24.8 (0.7) vs. 14.9 (0.7) ppb (p<0.01) and the greatest degree of airway hyperresponsiveness of the four clusters, with a PC_20_ FEV_1_ of 0.7 mg/mL methacholine. In both clusters, IgE and hsCRP were elevated when compared with non-obese clusters but were not significantly different from each other (p = 0.32 and 0.82, respectively). Thus, while obese individuals shared similar degrees of lung function impairment, adipokines, atopy and systemic inflammation (as indicated by hsCRP), a more severe group could be identified that had asthma of childhood onset, greater airway hyperresponsiveness, greater airway inflammation (as reflected by F_E_NO), and persistence of symptoms and suboptimal asthma control despite treatment with ICS.

### Characteristics of Non-obese Asthmatics

Non-obese clusters 1 and 2 differed from each other with regard to baseline lung function, with FEV_1%_ predicted of 87.7% (12.1) in cluster 1 and 82.3% (16.4) in cluster 2 (p = 0.02). A similar trend was seen with FEV_1_/FVC ratio, which was 74.1 (8.7)% in cluster 1 and 68.5 (8.7)% in cluster 2 (p<0.01). These two clusters also differed with regard to the percent of subjects who were male, at 18 vs. 83% (p<0.01) and age at asthma onset, at 19.1 (16.1) vs. 9.8 (11.8) years (p<0.01). Asthma symptom expression (AEQ scores of 0.5 (0.6) and 0.4 (0.5), p = 0.66) and degree of asthma control (ACQ scores of 0.8 (0.7) and 0.8 (0.6), p = 0.81) were similar between the two clusters, and these clusters were also similar with regard to biomarkers of inflammation (F_E_NO, IgE and hsCRP), indicating that the observed differences between clusters in lung function, sex, and age at disease onset were not linked with a distinct inflammatory phenotype (Table 3).

### Cluster Membership, BMI and *in vitro* GC Sensitivity

Markers of *in vitro* GC response were assessed in 49 participants in a single center translational mechanistic substudy. In members of obese clusters 3 and 4 (n = 12), PBMC GCRα expression (pg/ng 18 s RNA, log-transformed) was significantly less than in members of the non-obese clusters 1 and 2, at 6.6 (0.3) versus 6.9 (0.3), p = 0.004, corresponding with an approximately 25% reduction in the absolute values of GCRα expression in obese asthmatics (742.8 (184.5) vs. 984.7 (276.1) pg/ng 18 s RNA, p = 0.007).

When we analyzed the correlation between log-transformed GCRα expression in all 49 participants, we observed an inverse correlation (*r* = −0.23) that was not statistically significant (p = 0.1). Next, due to prior reports suggesting a relationship between vitamin D and biomarkers of steroid responsiveness [20], [21], we measured 25(OH)D concentrations in these participants. Members of clusters 3 and 4 demonstrated reduced 25(OH)D when compared with cluster 1 and 2 members, at 21.2 (7.6) vs. 29.2 (9.9) ng/mL (p = 0.01). We then analyzed the relationship between GCRαexpression and BMI in subjects who had 25(OH)D concentrations ≤30 ng/mL [20]. In this subset, an inverse correlation between BMI and log-transformed GCRαexpression was observed, with *r* = –0.52 (p = 0.02). This exploratory analysis suggested that the negative effect of BMI on GCRαexpression is augmented by 25(OH)D concentrations. Of note, 25(OH)D was not significantly correlated with GCRαexpression.

**Figure 1:**
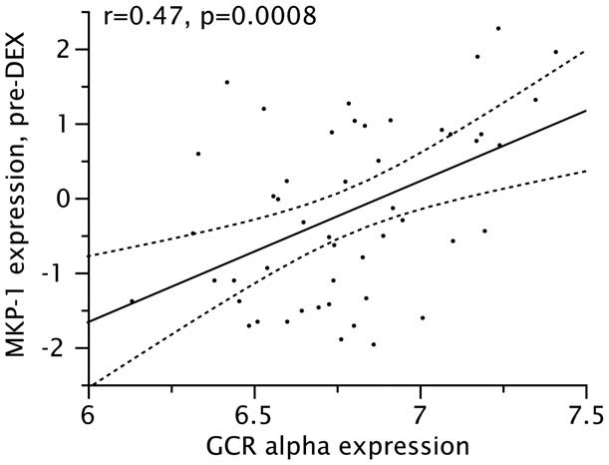
Correlation between expression of GCRα and baseline expression of MKP-1 in PBMC (both log-transformed).

**Figure 2:**
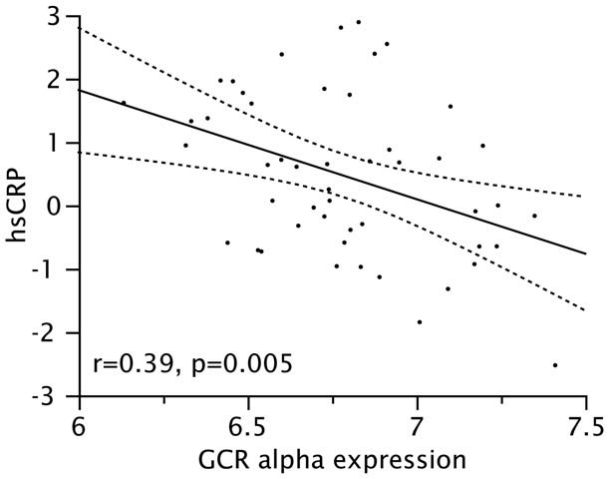
Correlation between expression of GCRα in PBMC and serum hsCRP concentrations of (both log-transformed).

Finally, to determine if reduced GCRαexpression might be one factor leading to reduced *in vitro* responsiveness to GCs reported in obese asthmatics [6], the correlation between GCRα expression (log-transformed) and MKP-1 expression both before and after exposure to dexamethasone was examined. Expression of GCRαwas significantly and positively correlated with baseline (pre-DEX, log-transformed) expression of MKP-1, with an unadjusted *r* = 0.47 (p = 0.008, Figure 1) and an *r* = 0.47 (p = 0.004) when adjusted for 25(OH)D concentrations. A significant positive correlation between GCRα and fold-induction of MKP-1 expression by DEX was also observed, with an adjusted *r* = 0.38 (p = 0.03). Additionally, concentrations of hsCRP, which were increased in obese cluster members, were found to be inversely correlated with GCRα expression, with an *r* = −0.39 (p = 0.005, Figure 2). Due to small sample size in clusters 3 and 4, we were unable to demonstrate a correlation between the differential clinical response to GC and *in vitro* markers of GC response in clusters 3 and 4.

## Discussion

The application of an hypothesis-free cluster analytical approach to a well-characterized cohort of adults with mild-to-moderate persistent asthma demonstrates that obesity is a determinant of clinical phenotype in asthma, playing a more significant role than other commonly-assessed clinical, physiologic or inflammatory variables. Of the four distinct clusters of asthma revealed, two had BMI in the obese range and two did not. There was heterogeneity of airway inflammation, symptoms and control in the obese clusters, suggesting that asthma phenotype is not uniform in obese individuals. In the two non-obese clusters, sex emerged as an important determinant of cluster membership; one cluster had a predominance of males the other a predominance of females, with comparatively earlier age of onset and lower lung function (as reflected by FEV_1%_ predicted) in the male-predominant cluster. Additionally, we have demonstrated that *in vitro* GC insensitivity in obese asthmatics (as represented by a reduced ability of dexamethasone to induce the expression of MKP-1, an anti-inflammatory marker of GC-induced transactivation [22]) appears to be mediated by reduced expression of GCRα, the dominant isoform of the receptor and a ligand-dependent transcription factor necessary for glucocorticoid-induced transactivation [23]. Exploratory analysis also suggests an important role for 25(OH)D concentrations in mediating this relationship.

Our findings also suggest that the mechanisms which underlie clinical response to GC in obese asthmatics are complex and likely involve an interaction between alterations in GC-mediated anti-inflammatory processes and both systemic and airway inflammation. This conclusion is based on our observation that while evidence of *in vitro* GC insensitivity was observed across both obese clusters, persistently poor asthma control and increased symptoms were observed in the cluster of asthmatics with the earliest onset of asthma, a greater degree of airway hyperresponsiveness and increased concentration of nitric oxide in exhaled breath. The GC insensitivity observed in obese asthmatics was also directly associated with the degree of systemic inflammation, as indicated by the inverse association between hsCRP and GCRα expression, and also is enhanced in the presence of reduced serum 25(OH) vitamin D concentrations. It is also interesting to note that our findings appear to minimize the role of comparative differences in sputum eosinophils as a reason for GC insensitivity in obese asthmatics. Independent of BMI, sputum eosinophils averaged less than 1% in the study population, suggesting that the GC insensitivity observed in obese patients with asthma is likely attributable to the defects in molecular GC response or increased inflammation that we have demonstrated, rather than to a pauci-eosinophilic airway inflammatory phenotype specific to obese asthmatics, as has been suggested in other reports [1].

Potential limitations of our must be considered: first, our analytical approach is hypothesis-independent. While this provides the opportunity to identify new associations that one might not be able (on the basis of current knowledge) to prespecify, it runs the risk of returning results that are counterintuitive or which differ from current hypothetical constructs of disease. Second, as with any meta-analytical technique, the results are entirely dependent on the data available for entry into the analysis. Thus, while we have attempted to include all clinically-relevant data, the derivation of our data from a clinical trial dataset limits the availability of certain data (e.g. socioeconomic or environmental status) and may introduce issues of generalizability given the highly-selected nature of clinical trial participants. Next, as with any cross-sectional data, we are unable to comment on causation, *per se*, and thus can only conclude that there are specific aspects of the obesity-asthma relationship that are clinically relevant. In fact, a number of questions regarding causal aspects of the relationship between obesity and asthma remain unanswered. Although many epidemiologic studies suggest that antecedent obesity increases subsequent asthma risk, asthma could also increase the risk of becoming overweight or obese. Factors that may play a role in this regard include chronic glucocorticoid use leading to weight gain, as well as respiratory impairment leading to sedentariness, reduced participation in physical, educational or occupational activities, as well as overall reductions in quality of life, all of which may lead to or be associated with increases in body mass.

Our analytical approach and validation of clinical phenotypes with studies of the molecular mechanisms of GC insensitivity in asthma strengthen the assertion that patients with asthma, both adult and pediatric [3], [5], [24]–[27], who are overweight or obese bear a disproportionate burden of illness when compared with non-obese asthmatics. Notwithstanding, the mechanisms of GC insensitivity are complex [28], and the mechanisms by which obesity reduces molecular response to GC require further study. In this light, recent studies have shown that monokines secreted by adipose tissue activate blood monocytes and recruit activated macrophages to adipose tissue, significantly amplifying pro-inflammatory cytokine generation [29]–[32]. This phenomenon can be associated with classical activation [32] which has been documented in both blood monocytes and alveolar macrophages in glucocorticoid-insensitive asthma [33], [34], and may be relevant in obese patients with asthma as well. Additionally, these data provide additional support to the importance of recent observations [6], [21], [35]–[38], that low serum vitamin D concentrations are associated with impaired glucocorticoid response in asthma.

Clinicians frequently encounter obese asthmatics who do not respond optimally to therapy, but no specific guidance currently exists in national and international guidelines as to the optimal therapeutic approach to the obese asthmatic [39], [40]. Many obese asthmatics receive complicated asthma treatment regimens to which they do not respond [2] and which may subject them to increased risk of treatment-related adverse effects. Given this, and given the prevalence of both obesity and asthma, additional information on which to base therapeutic decision making is critical. This study used unbiased analytical approaches to further validate reports that asthma phenotype differs between obese and non-obese asthmatics, while also demonstrating that asthma phenotype is not homogenous in all obese individuals, particularly with regard to the degree of control achieved with ICS treatment. Thus, clinical GC response may not be uniformly attenuated in obese asthmatics, and more work is needed to identify the pathways by which GC signaling mechanisms, systemic inflammation and airway inflammation interact to lead to clinical insensitivity to GC in some, but not all, obese asthmatics.

## Supporting Information

Appendix S1 The following Asthma Clinical Research Network sites and investigators participated in the parent clinical trials which obtained the clinical data analyzed in this study.
(DOCX)
