# Supplementary material for: Cluster Analysis of Obesity and Asthma Phenotypes
Source: PLoS One. 2012 May 11;7(5):e36631. doi: 10.1371/journal.pone.0036631 (PMC3350517; doi:10.1371/journal.pone.0036631)
Supplement: Appendix S1 — The following Asthma Clinical Research Network sites and investigators participated in the parent clinical trials which obtained the clinical data analyzed in this study. (DOCX) [file pone.0036631.s001.docx]

*Brigham & Women's Hospital, Boston, MA*: E. Israel, M. E. Wechsler

*Columbia University, New York, NY*: E. DiMango

*Duke University, Durham, NC:* M. Kraft*,* N. L. Lugogo

*National Jewish Health, Denver, CO*: R. J. Martin, R. M. Cherniack, S. J. Szefler, E. R. Sutherland

*Pennsylvania State College of Medicine, Hershey, PA*: V. M. Chinchilli, T. J. Craig, N. Icitovic, T. S. King, S. J. Kunselman, E. A. Mauger, L. L. Engle

*University of California, San Diego Medical Center, CA*: J. Ramsdell, S. I. Wasserman

*University of California, San Francisco, CA*: H. A. Boushey, J. V. Fahy, S. C. Lazarus

*University of Texas Medical Branch, Galveston, Tex*: W. J. Calhoun, B. T. Ameredes

*University of Wisconsin School of Medicine and Public Health, Madison, WI*: R. F. Lemanske, C. A. Sorkness, N. N. Jarjour, L. Denlinger.

*Wake Forest University, School of Medicine, Winston-Salem, NC*: E. Bleecker, D. Meyers, S. P. Peters, W. C. Moore, R. Pascual

*Washington University, School of Medicine, St Louis, MO*: M. Castro, M. J. Walter
